# Diversity of Subgroup of *Fibrobacter succinogenes* in the Rumen and Feces of Cattle and Sheep

**DOI:** 10.1111/asj.70043

**Published:** 2025-02-25

**Authors:** Chiaki Ogawa, Tomomi Ban‐Tokuda, Hiroki Matsui

**Affiliations:** ^1^ Graduate School of Bioresources Mie University Tsu Mie Japan

**Keywords:** feces, *Fibrobacter succinogenes*, rumen fluid, rumen mat, ruminants

## Abstract

*Fibrobacter succinogenes* is the most important microbe in terms of fiber degradation in the rumen of ruminants and is known to be a phylogenetically diverse group of species. This study aimed to examine the ecology and diversity of *F. succinogenes* in the rumen and hindgut of cattle and sheep using a species‐specific primer set. Using this technique, six clone libraries were constructed, and the cloned sequences were analyzed. FS I was dominated by cattle rumen fluid (CRF), cattle rumen mat (CRM), sheep rumen fluid (SRF), and sheep rumen mat (SRM). FS II was dominated by cattle feces (CFE). FS III was dominated by sheep feces (SFE). FS III and IV were commonly detected in both CFE and SFE. Horse and capybara group was detected in SFE. Abundance of *F. succinogenes* was determined by species specific real‐time PCR assay. The abundance in the rumen mat was significantly higher than in the rumen fluid and feces in cattle (*p* < 0.01). In conclusion, the composition of the *Fibrobacter* group in the rumen was different from that in the feces, indicating that the composition in the feces does not directly reflect the composition of the rumen.

## 1 Introduction

Ruminants ingest plant particles as feed into the rumen. The rumen microbial ecosystem is populated by a diverse collection of obligately anaerobic microorganisms, including bacteria, protozoa, fungi, and methanogenic archaea (Tajima et al. 1999). The majority of the fibers in the plant cell walls are cellulose and hemicellulose. The process of breaking down recalcitrant polysaccharides in the plant cell walls into soluble sugars is carried out by fibrolytic microorganisms in the rumen. They produce a variety of enzymes that break down polysaccharides, including cellulases, hemicellulases, and pectinases. It is suggested that the cellulolytic bacterium *Fibrobacter succinogenes* is the most important microbe in terms of ruminal fiber degradation (Cheng et al. 1983; Miron 1991).

*F. succinogenes* is a nonmotile, strictly anaerobic, Gram‐negative, and rod‐shaped bacterium. This bacterium is known as one of the most active cellulolytic bacteria (Bryant 1954; Halliwell and Bryant 1963) and is known as one of the predominant fibrolytic bacteria in the rumen (Bera‐Maillet et al. 2004; Weimer et al. 1991; Lwin et al. 2012). The number of the bacterium is much higher in the rumen of the animals fed high forage diet (Tajima et al. 2001). *F. succinogenes* is known to be a phylogenetically diverse group of bacterium. Recently, *F. succinogenes* was divided into seven groups according to phylogenetic positioning of near full length of 16S rRNA gene sequences of the isolates (Neumann et al. 2017). However, none of the studies has been conducted to survey on ecology of the group in the hindgut of ruminants.

In the present study, PCR detection of *F. succinogenes* from feces or rumen content (both solid phase and liquid phase) of cattle and sheep using species‐specific primers was carried out. Clone libraries were constructed from the amplified DNA fragments, and cloned sequences were phylogenetically analyzed. Furthermore, quantitative analysis of *F. succinogenes* population was assessed by species‐specific real‐time PCR.

## 2 Materials and Methods

### 2.1 Animal and Feeding

Animal handling was performed according to the Animal Handling Guidelines of the Graduate School of Bioresources, Mie University. Three ruminally cannulated crossbreed cattle (Holstein × Japanese Black Cattle) and sheep were used in this study. The average bodyweight of the cattle and the sheep were 363.3 ± 11.0 kg and 35.7 ± 3.1 kg, respectively. The animals were fed Italian ryegrass straw and commercial concentrate (Soyokaze no Kaori; Nippon Formula Feed Manufacturing, Yokohama, Japan). The concentrate consisted of 44% corn grain, 44% cereal mix, 27% wheat bran and rice bran, 19% plant oil meal, and 10% pellet mineral mix containing 18.0% crude protein, 4.0% crude fat, 7.1% crude fiber, 6.7% crude ash, 0.60% calcium, and 0.80% phosphorous (dry matter basis). The total digestible nutrient in the concentrate was 82.9%, and no antimicrobial agents were incorporated in the diet. Cattle were given 4.0 kg day^−1^ each of the forage and the concentrate. Sheep were given 600 g day^−1^ of the forage and 200 g day^−1^ of the concentrate. The diet was divided equally and provided twice a day at 10.00 and 17.00 h. Water and mineral blocks were available ad libitum.

### 2.2 Sample Collection

Rumen contents including the solid phase (rumen mat) and fluid phase were collected via cannula just before the morning feed. Solid phase was obtained by squeezed with four layers of surgical gauze. Liquid phase was aspirated through a polyvinyl chloride pipe and filtered through with four layers of surgical gauze. Feces in the rectum were taken by wearing disposable plastic gloves just prior to the morning feed. The samples were immediately put on ice and transferred to the laboratory. They were stored at −80°C until analysis.

### 2.3 DNA Extraction

Microbial DNA in the rumen contents and feces was extracted using a QIAamp DNA Stool kit (Qiagen, Hilden, Germany) according to the manufacturer's protocol with bead beating with FastPrep FP100A instrument (MP Biomedical, Sant Ana, CA, United States). The purity and concentration of DNA samples were determined using NanoDrop™ 2000 Spectrophotometers (Thermo Scientific™), and all samples were adjusted to have a final concentration of 10 ng μL^−1^ with an A260/A280 ratio of > 1.8. Extracted DNA was stored at −80°C until further analysis.

### 2.4 Construction of Clone Library and Sequence Analysis

Microbial DNA from solid and fluid fractions of rumen and feces of each individual animal was used as template DNA for the construction of clone libraries. A clone library of partial 16S ribosomal RNA gene (16S rDNA) of *F. succinogenes* was constructed as previously described by Matsui et al. (2010). Sequences forming chimera were determined by the DECIPHER program (Wright et al. 2012). Operational taxonomic unit (OTU) and clustering were calculated using the “mothur” program Version 1.36.1 (Schloss et al. 2009). DNA sequences were assigned to individual OTUs based on a 99% sequence similarity criterion according to a previous report (Lwin et al. 2012). Assignment of each sequence to genera was performed using RDP Naïve Bayesian rRNA Classifier version 2.10 (RDP Release 11, Update 4) (Wang et al. 2007). The DNA sequences were aligned with MUSCLE (Edgar 2004) using MEGA6 (Tamura et al. 2013), and a phylogenetic tree was constructed using the neighbor‐joining method (Saitou and Nei 1987). Bootstrapping (1000 resamplings) was used to estimate the confidence of the branch patterns. Unweighted pair group method with arithmetic mean (UPGMA) was calculated using the “mothur” program.

### 2.5 Real‐Time PCR Assay

Real‐time PCR assay was performed using Thunderbird® SYBR® qPCR Mix (TOYOBO, Osaka, Japan) and a StepOne Plus™ Real‐Time PCR Systems (Applied Biosystems, Foster City, CA, United States). A primer pair Fsf and Fsr (Matsui et al. 2010) was used to quantify the 16S rDNA of *F. succinogenes* . The purified DNA was used as a template. The reaction mixture (20 μL) contained 1.0‐μL DNA, 10.0‐μL Thunderbird® SYBR® qPCR Mix, 0.9 μM of each primer, and 0.4‐μL 50 × ROX. The 16S rDNA of total bacteria was determined as described by Arfan et al. (2016). The reaction mixture (20 μL) contained 1.0‐μL DNA, 10.0‐μL Thunderbird® SYBR® qPCR Mix, 0.15 μM of each primer, and 0.4‐μL 50 × ROX. Both assays were carried out under the following conditions: activation of polymerase at 95°C for 1 min, followed by 40 cycles of 95°C for 15 s, 60°C for 1 min. Amplicon specificity was determined via dissociation curve analysis of PCR end products by increasing the temperature from 60°C to 95°C at a rate of 1°C/30 s. The calculation was performed using StepOne™ Software 2.2.

### 2.6 Statistical Analysis

Population density of *F. succinogenes* was statistically analyzed using SPSS ver.27. Data were subjected to one‐way ANOVA. Tukey significant difference method was used as a post hoc test. Differences of *p* < 0.05 were considered significant, and differences of *p* < 0.01 were considered highly significant.

### 2.7 Sequence Accession Numbers

All nucleic acid sequences obtained in this study were deposited in the DDBJ, EMBL, and GenBank databases under accession numbers LC137027 to LC137281 for cattle rumen fluid library, LC137284 to LC137362 for sheep rumen fluid library, LC137363 to LC137440 for cattle rumen mat library, LC137440 to LC137521 for sheep rumen mat library, LC137522 to LC137601 for cattle feces, and LC137602 to LC137681 for sheep feces.

## 3 Results

A total of 475 sequences obtained from libraries were analyzed. No chimera sequence was found in all libraries. The number of clones analyzed in each library ranged between 77 and 81. These sequences were classified into OTU with a criterion of 99% homology.

**FIGURE 1:**
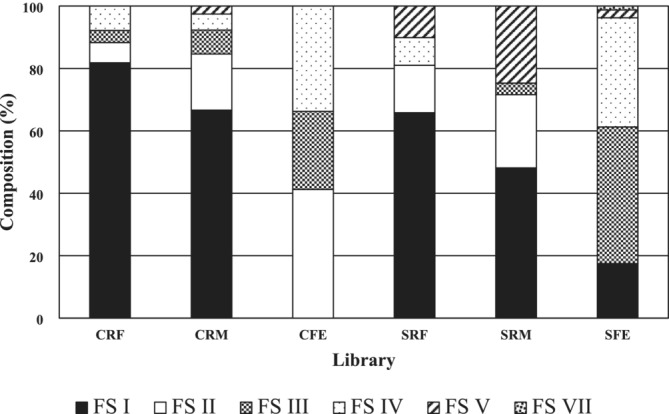
Composition of each group within *Fibrobacter succinogenes* in each library recovered from rumen solid and fluid phase and feces of cattle and sheep. Groups of *F. succinogenes* are based on Neumann et al. (2017). CFE, cattle feces; CRF, cattle rumen fluid; CRM cattle rumen mat; SFE, sheep feces; SRF, sheep rumen fluid; SRM, sheep rumen mat.

The cloned sequences were grouped into FS I, FS II, FS III, FS IV, FS V, and FS VII according to the phylogenetic positioning of each sequence as described in Neumann et al. (2017) (data not shown). The composition of each group within *F. succinogenes* in each library is shown in Figure 1. FS I was dominated by cattle rumen fluid (CRF), cattle rumen mat (CRM), sheep rumen fluid (SRF), and sheep rumen mat (SRM). Subsequently, FS II was dominated by cattle feces (CFE). FS III, IV, and V were minor components among CRF, CRM, and SRF. In SRM, the share of FS V was 25%. The composition of the group was different in CFE and sheep feces (SFE). FS II was the most dominant in CFE. FS IV was the second most abundant in CFE. Although FS III was minor in rumen contents (CRF, CRM, SRF, and SRM), it was major in CFE. The most abundant group was FS III in SFE, followed by FS IV. Like CFE, FS IV was the second most abundant group in SFE. Interestingly, FS VII was only detected in SFE.

**FIGURE 2:**
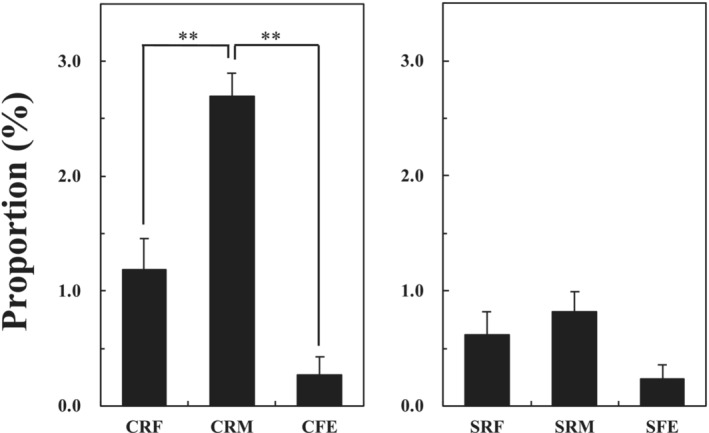
Relative abundance of *Fibrobacter succinogenes* against total bacteria in the rumen solid and fluid phase and feces of cattle and sheep determined by species‐specific real‐time PCR assay. CFE, cattle feces; CRF, cattle rumen fluid; CRM cattle rumen mat; SFE, sheep feces; SRF, sheep rumen fluid; SRM, sheep rumen mat. **Statistically significant between samples (*p* < 0.01).

Relative abundance (%) of *F. succinogenes* against total bacteria in each fraction was determined by real‐time PCR assay (Figure 2). The abundance of *F. succinogenes* in CRM was significantly higher than the CRF and CFE (*p* < 0.01). There was no significant difference in the abundance of *F. succinogenes* in the SRF, SRM, and SFE. The abundance in the feces was the lowest in both cattle and sheep. The *F. succinogenes* population in the CFE was 1/10 of the CRM.

**FIGURE 3:**
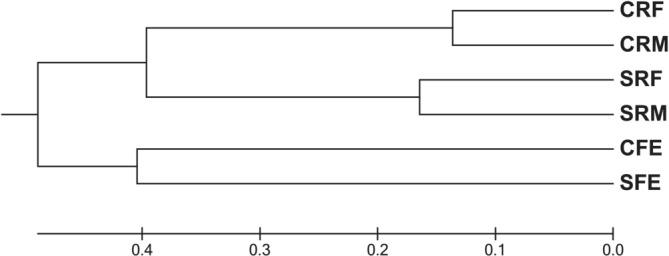
Dendrogram showing similarity between each library constructed from rumen contents and feces of cattle and sheep. CFE, cattle feces; CRF, cattle rumen fluid; CRM cattle rumen mat; SFE, sheep feces; SRF, sheep rumen fluid; SRM, sheep rumen mat.

Dendrogram generated with an unweighted pair group method with arithmetic mean (UPGMA) is shown in Figure 3. CRF and CRM were clustered together. Similarly, SRF and SRM were clustered. CFE and SFE were distantly clustered.

## 4 Discussion

Lin et al. (1994) monitored *F. succinogenes* in the rumen using group‐specific oligonucleotide probes and reported that Groups 1 and 2 were detected from the rumen of steers and goats. On the other hand, Koike et al. (2004) reported that only Groups 1 and 3 were detected in the rumen of wethers and steers. Shinkai et al. (2007) showed that Groups 1–3 were detected in the rumen of sheep and cows. In contrast to these reports, our results demonstrated that FS IV (syn. Group 4) and FS V were also detected in addition to FS I to III (syn. Groups 1–3) in the rumen of cattle and sheep (Figure 1). Neumann et al. (2017) isolated 39 novel *F. succinogenes* strains from nine different animals. These strains were divided into seven distinct groups. Interestingly, six of these groups were detected in the rumen and feces of cattle and sheep in the present study (Figure 2). Thus, ruminants harbor most of the *F. succinogenes* groups. They also suggested that FS IV (syn. Group 4) was widely distributed to many host animals. FS IV was detected in the rumen and the feces of cattle and sheep in this study (Figure 2). Our results support their observation. Neumann et al. (2017) failed to isolate the FS V group from ruminants. However, the group was detected in the CRM, SRF, SRM, and SFE in this study.

The ability of the *F. succinogenes* strains to digest forage materials differed from the phylogenetic group of the *F. succinogenes* (Shinkai et al. 2009). The diversity of groups of *F. succinogenes* in the rumen would be affected by the types of forages ingested by the host animals. Substrates available in the rumen and large intestine are different. Intact leaves and stems of forages are ingested into the rumen. Therefore, *F. succinogenes* and other fibrolytic microorganisms can utilize the intact plants. In the intact plant cell wall, cellulose is covered with hemicellulose and lignin. It is essential to remove hemicellulose and lignin to expose and utilize cellulose fraction for degradation and fermentation by *F. succinogenes* . Recalcitrant substrates that escaped from the rumen degradation are available in the large intestine. *F. succinogenes* FS III or IV abundant in the feces (Figure 2) may utilize such substrates and survive in the large intestine.

UPGMA analysis showed that CRF and CRM, and SRF and SRM were clustered (Figure 3). Furthermore, CFE and SFE were distantly related to these fractions. Comparative analysis of whole genomes from 38 novel *Fibrobacter* strains against the type strains for the two formally described *Fibrobacter* species *F. succinogenes* S85 and *F. intestinalis* NR9 was carried out (Neumann and Suen 2018). They found that genomes of *F. succinogenes* phylotypes that are dominant in the rumen had significantly more genes annotated to major families involved in hemicellulose degradation (e.g., CE6, GH10, and GH43) than did the genomes of *F. succinogenes* phylotypes typically observed in the lower gut of large hindgut‐fermenting herbivores such as horses.

They also find that genes encoding a putative urease were also identified in 12 of the *Fibrobacter* genomes, which were primarily isolated from hindgut‐fermenting hosts. These genomic differences would reflect the composition of *Fibrobacter* groups in the rumen and feces of the ruminants examined in this study. Sugars are generally considered to be a carbohydrate fraction that ferments very quickly in the rumen (Oba 2011). Therefore, sugar concentration is very low in the large intestine. Sugars available to *F. succinogenes* would be large in the rumen compared with the large intestine. These differences in the concentration of sugars between the rumen and large intestine may cause differences in the compositions of *F. succinogenes* . Moreover, there is a difference in the outflow rate from the rumen and large intestine in the sheep (de Vega et al. 1998). The outflow rate is higher in the large intestine compared to the rumen. Hence, only the *Fibrobacter* group with a faster growth rate could survive in the large intestine.

Michalet‐Doreau et al. (2001) reported that the population size of *F. succinogenes* was significantly higher in the solid phase than in the liquid phase of rumen contents. Our results also support this observation (Figure 2).

In conclusion, the composition of the *Fibrobacter* group in the rumen was different from that in the feces, indicating that the composition in the feces does not directly reflect the composition of the rumen. This indicates that *Fibrobacter* group composition changes in response to environmental conditions.

## Conflicts of Interest

The authors declare no conflicts of interest.
